# PCBert-Kla: an efficient prediction method for lysine lactylation sites based on ProtBert and fusion of physicochemical features

**DOI:** 10.1093/bib/bbaf615

**Published:** 2025-11-19

**Authors:** Hong-Qi Zhang, Yi-Xuan Qi, Huma Fida, Hao-Jiang Zhang, Muhammad Arif, Pei-Yu Zhao, Tanvir Alam, Ye-Chen Qi, Xiao-Long Yu, Ke-Jun Deng

**Affiliations:** School of Life Science and Technology and Center for Informational Biology, University of Electronic Science and Technology of China, No. 2006 Xiyuan Avenue, West Hi-Tech Zone, Chengdu 610054, China; School of Life Science and Technology and Center for Informational Biology, University of Electronic Science and Technology of China, No. 2006 Xiyuan Avenue, West Hi-Tech Zone, Chengdu 610054, China; School of Life Science and Technology and Center for Informational Biology, University of Electronic Science and Technology of China, No. 2006 Xiyuan Avenue, West Hi-Tech Zone, Chengdu 610054, China; School of Life Science and Technology and Center for Informational Biology, University of Electronic Science and Technology of China, No. 2006 Xiyuan Avenue, West Hi-Tech Zone, Chengdu 610054, China; College of Science and Engineering, Hamad Bin Khalifa University, Education City, Doha 34110, Qatar; School of Computer Science and Technology, Hainan University, No. 58 Renmin Avenue, Meilan District, Haikou 570228, China; College of Science and Engineering, Hamad Bin Khalifa University, Education City, Doha 34110, Qatar; School of Life Science and Technology and Center for Informational Biology, University of Electronic Science and Technology of China, No. 2006 Xiyuan Avenue, West Hi-Tech Zone, Chengdu 610054, China; School of Materials Science and Engineering, Hainan University, No. 58 Renmin Avenue, Meilan District, Haikou 570228, China; School of Life Science and Technology and Center for Informational Biology, University of Electronic Science and Technology of China, No. 2006 Xiyuan Avenue, West Hi-Tech Zone, Chengdu 610054, China

**Keywords:** post-translational modifications, lysine lactylation, ProtBert, deep learning, feature fusion

## Abstract

Protein post-translational modifications (PTMs) play a critical role in regulating protein functionality and structural diversity. Among them, lysine lactylation (Kla), a newly identified PTM, is involved in energy metabolism, cellular reprogramming, and the progression of various diseases. In this study, we propose PCBert-Kla, a feature-fusion deep learning model based on ProtBert. This model leverages ProtBert to extract deep features from protein sequences, effectively capturing global and local contextual information. It integrated various physicochemical properties, including molecular weight, isoelectric point, amino acid composition, secondary structure content, hydrophobicity, and net charge. An attention mechanism in the fully connected layers enabled the model to select features automatically. PCBert-Kla exhibited exceptional accuracy and reliability in Kla site identification and demonstrated excellent generalization capability to outperform the existing models. In addition, we further enhanced the interpretability of the PCBert-Kla model by incorporating average attention maps. This model provided powerful tools for studying the functions of Kla and elucidating the mechanisms of related diseases, which can advance biomedical research and drug development. We also developed a free web service, available at http://pcbert-kla.lin-group.cn/, to provide users with easy access and usage.

## Introduction

Protein post-translational modifications (PTMs) refer to covalent modifications of specific amino acid residues after mRNA translation into proteins [1–5]. This process serves as a crucial mechanism for increasing functional and structural diversity [6, 7]. Recent research has identified a novel PTM—lysine lactylation (Kla)—which involves a lactyl group at the Nε-position of lysine residues [8]. Kla regulates critical cellular functions, including energy metabolism, cellular reprogramming, tissue repair, neural excitation, Alzheimer’s disease, inflammatory responses, and immune suppression [9–12]. The discovery of Kla highlights its essential role in biological activities and diseases, particularly in metabolic regulation associated with the Warburg effect [13]. However, accurately identifying Kla sites remains a significant challenge.

Identifying Kla sites primarily relies on high-performance liquid chromatography-tandem mass spectrometry (HPLC-MS/MS) [9, 14]. Although the experimental approach can directly detect Kla sites, its difficulty, high cost, and low throughput limit its widespread application [15]. Consequently, computational methods have emerged as alternative or complementary approaches for Kla site identification [16–22]. The existing computational methods for Kla site identification were represented primarily by DeepKla and Auto-Kla [23, 24]. DeepKla was the first computational framework developed for Kla site identification. It leveraged deep learning models such as embedding layers, convolutional neural networks (CNNs) [25–28], bidirectional gated recurrent units, and attention mechanisms, which automatically extract features from protein sequences and perform end-to-end predictions [29–32]. Auto-Kla, on the other hand, is a web-based service built on automated machine-learning techniques. It integrates the Transformer deep language model with automated machine learning to develop robust and generalizable PTM site prediction models. While these methods have made significant progress in Kla site prediction, several challenges remain: (i) the performance of current models still has room for improvement; (ii) the impact of traditional bioinformatics feature extraction on the feature space of deep learning models has not been extensively studied; (iii) the application of protein large language models in this domain remains underexplored. These limitations highlight the need for further advancements in Kla site prediction methodologies.

We propose PCBert-Kla, a computational model for efficient and accurate prediction of Kla sites. PCBert-Kla was constructed based on the ProtBert pretrained protein language model, which effectively extracts deep features from protein sequences and captures global and local contextual information, generating high-quality sequence representations. It integrated physicochemical properties such as molecular weight, isoelectric point, amino acid composition, secondary structure content, hydrophobicity, and net charge. The integrated feature space overcame the limitations of using sequence-based features alone [33]. Furthermore, we designed an improved attention-based fully connected layer as the classifier, enabling dynamic focus on key features associated with Kla modifications, thereby improving prediction accuracy and robustness.

## Data and methods

### Data

The dataset used in this study includes Kla site sequences and their corresponding non-Kla site sequences. We directly used the benchmark dataset proposed by Lv *et al.* To ensure a fair comparison, no modifications were made to the data. The dataset is publicly available for download at https://github.com/linDing-group/DeepKla [24]. Lv *et al.*’s data collection and processing procedure is as follows: Rice Kla data were collected from relevant literature and used as training samples. Lysine sites annotated as lactylated were considered positive samples, whereas lysine residues in the same protein that were not annotated as lactylated were regarded as negative samples. Additionally, a protein sequence length of 51 was chosen, with lysine sites located in the center, and the CD-HIT [34, 35] threshold was set to 30%. The training set consists of 1720 Kla sequences and 1767 non-Kla sequences, while the independent external test set contains the same number of 177 Kla sequences and 177 non-Kla sequences (Table S1).

### Methods

**Figure 1:**
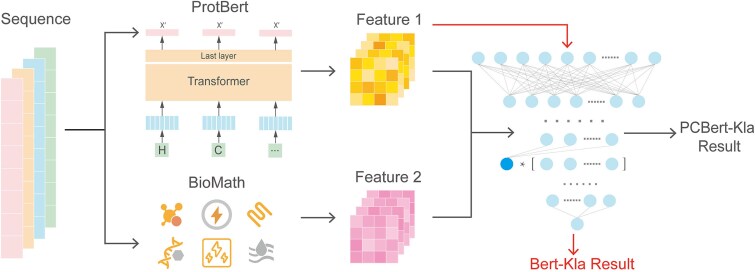
Model architecture diagram.

PCBert-Kla used a pretrained protein large language ProtBert model to extract deep features from protein sequences. Physicochemical properties [36, 37], including molecular weight, isoelectric point, amino acid composition, secondary structure content, hydrophobicity, and net charge, were incorporated to further enrich the feature space. Furthermore, we designed an improved attention-based fully connected layer as the classifier for the PCBert-Kla (Fig. 1). In addition, the graphics processing unit (GPU) resources used in this study were the NVIDIA GeForce RTX 4090.

#### ProtBert

Bidirectional encoder representations from transformers (BERT) is a pretrained language model based on the Transformer architecture, excelling in natural language processing (NLP) and providing a strong foundation for a wide range of language understanding tasks [38]. The key feature of BERT lies in its ability to capture bidirectional contextual information, i.e. it considers both forward and backward context simultaneously, enabling superior performance across various NLP tasks. Previous models had achieved remarkable results in pretrained language modeling but were typically unidirectional, leveraging only forward or backward context [39]. This limitation hindered their capability to understand global textual context. BERT overcame this limitation by introducing two novel training tasks: masked language model (MLM) and next sentence prediction (NSP). MLM: randomly masks portions of the input text and trains the model to predict the masked tokens, thereby learning bidirectional context representations. NSP: enhances the model’s understanding of relationships between sentences by predicting whether one sentence follows another. Through pretraining on massive unlabeled datasets and fine-tuning downstream tasks, BERT significantly improved NLP task performance and became a groundbreaking advancement in the field [40–42].

Rostlab/prot_bert (ProtBert) is a protein-specific language model built on the BERT architecture, pretrained via self-supervised learning on the UniRef100 dataset, which contains 217 million protein sequences. Unlike the original BERT, ProtBert treats each sequence as an independent ``document'' and separates them with blank lines. This means it does not use NSP, as each sequence is regarded as a complete document, further optimizing its ability to process sequences. The ProtBert model generates 1024-dimensional feature vectors for each protein sequence subsequently used to predict Kla sites. Additionally, when ProtBert was integrated into PCBert-Kla, only the first four layers of the BertEncoder were retained, and the remaining layers were removed. The retained layers were not frozen; instead, the entire architecture was fine-tuned during training.

#### Molecular weight calculation

In this study, the protein sequence mass is calculated using methods provided by Biopython. Specifically, this approach first obtains the mass of each amino acid using a standardized amino acid mass table and sums the masses of all amino acids. Then, the mass of the water molecule is subtracted to obtain the actual mass of the protein. The final feature extraction result is a one-dimensional value. The calculation method is as follows:


(1) \begin{equation*} \boldsymbol{M}{\boldsymbol{W}}_{\boldsymbol{protein}}=\sum_{\boldsymbol{i}=\mathbf{1}}^{\boldsymbol{n}}{\boldsymbol{MW}}_{\boldsymbol{A}{\boldsymbol{A}}_{\boldsymbol{i}}}-\left(\boldsymbol{n}-\mathbf{1}\right)\times{\boldsymbol{MW}}_{{\boldsymbol{H}}_{\mathbf{2}}\boldsymbol{O}} \end{equation*}

where $M{W}_{protein}$represents the molecular weight of the protein, ${MW}_{A{A}_i}$denotes the molecular weight of the $i$-th amino acid, $n$ represents the total number of amino acids in the protein sequence, and ${MW}_{H_2O}$represents the molecular weight of a water molecule.

#### Isoelectric point calculation

This study directly uses the methods provided by Biopython. The specific implementation involves iteratively calculating the protein's charge at different pH values using a bisection method, until a pH value is found where the charge is close to zero, which corresponds to the isoelectric point. Ultimately, the feature extraction results in a one-dimensional value.

#### Amino acid composition analysis

Amino acid composition analysis is used to calculate the relative percentage of each amino acid in a protein sequence [29, 43]. Ultimately, this method can output a 20-dimensional feature. The calculation method is as follows:


(2) \begin{equation*} {\boldsymbol{A}\boldsymbol{A}}_{{\boldsymbol{percent}}_{\boldsymbol{i}}}=\frac{{\boldsymbol{Count}}_{\boldsymbol{A}{\boldsymbol{A}}_{\boldsymbol{i}}}}{\boldsymbol{n}}\times \mathbf{100}\% \end{equation*}

where ${AA}_{percent_i}$represents the percentage of the $i$-th amino acid in the protein sequence, ${Count}_{A{A}_i}$represents the number of occurrences of the $i$-th amino acid in the sequence, and $n$represents the total number of amino acids in the protein sequence.

#### Secondary structure content prediction

This study directly utilizes the methods provided by Biopython, which are based on the classification of amino acids according to their structural tendencies. The method estimates the potential composition of secondary structures by calculating the proportion of each amino acid category. Specifically, the estimation of the helix structure is based on the proportion of amino acids glutamic acid (E), methionine (M), alanine (A), leucine (L), and lysine (K), the turn structure is estimated based on the proportion of amino acids asparagine (N), proline (P), glycine (G), serine (S), and aspartic acid (D), and the sheet structure is estimated based on the proportion of amino acids valine (V), isoleucine (I), tyrosine (Y), phenylalanine (F), tryptophan (W), leucine (L), and threonine (T). The final feature extraction result is a three-dimensional value. Mathematically, this can be represented as:


(3) \begin{equation*} \boldsymbol{aa}\_\boldsymbol{proportions}\left[\boldsymbol{AA}\right]=\frac{\boldsymbol{count}\left(\boldsymbol{AA}\right)}{\ \mathrm{sequence}\ \mathrm{length}\ } \end{equation*}

(4) \begin{equation*} \boldsymbol{helix}={\sum}_{\boldsymbol{r}\in \boldsymbol{EMALK}}\kern0.1em \mathrm{aa}\_\mathrm{proportions}\ \left[\mathrm{r}\right] \end{equation*}

(5) \begin{equation*} \boldsymbol{turn}={\sum}_{\boldsymbol{r}\in \boldsymbol{NPGSD}}\mathrm{aa}\_\mathrm{proportions}\ \left[\mathrm{r}\right] \end{equation*}

(6) \begin{equation*} \boldsymbol{sheet}={\sum}_{\boldsymbol{r}\in \boldsymbol{VIYFWLT}}\mathrm{aa}\_\mathrm{proportions}\ \left[\mathrm{r}\right] \end{equation*}

where *count(AA)* is the number of occurrences of a specific amino acid in the sequence, and sequence length is the total length of the protein sequence.

#### Hydrophobicity calculation

This study directly uses the methods provided by Biopython, which are based on the Kyte–Doolittle hydropathy scale. The analysis is performed by calculating the average hydropathy values of the amino acids in the protein sequence. Ultimately, the feature extraction results in a one-dimensional data set.


(7) \begin{equation*} \mathrm{GRAVY}=\frac{\mathbf{1}}{\boldsymbol{L}}\sum_{\boldsymbol{aa}\mathbf{\in}\boldsymbol{sequence}\ }\kern0.1em \mathrm{selected}\_\mathrm{scale}\ \left[\mathrm{aa}\right] \end{equation*}

here, selected_scale[aa] is the hydropathy value of the amino acid aa (Kyte–Doolittle). sequence is the protein sequence, and L is the total length of the protein sequence.

#### Net charge calculation

This study directly uses the methods provided by Biopython. In this approach, the charge of each charged amino acid residue is calculated using a formula derived from the Henderson–Hasselbalch equation. For positively charged amino acids, their charge is calculated using the following formula:


(8) \begin{equation*} \boldsymbol{positive}\ \boldsymbol{charge}=\frac{\mathbf{1}}{\mathbf{1}+{\mathbf{1}\mathbf{0}}^{\left(\boldsymbol{pH}-\boldsymbol{pK}\right)}} \end{equation*}

For negatively charged amino acids, their charge is calculated using the following formula:


(9) \begin{equation*} \boldsymbol{negative}\ \boldsymbol{charge}=\frac{\mathbf{1}}{\mathbf{1}+{\mathbf{1}\mathbf{0}}^{\left(\boldsymbol{pK}-\boldsymbol{pH}\right)}} \end{equation*}

Ultimately, the net charge of the protein is the difference between the total positive and negative charges. The feature extraction results in a one-dimensional value.

#### Attention-based improved fully connected layer

In this study, we designed an attention-based improved classifier for predicting Kla sites in proteins. First, we extracted a 1024-dimensional feature vector [the feature of (CLS)] from the protein sequences using the ProtBert model. Additionally, we utilized traditional feature extraction techniques to derive a 27-dimensional feature vector, which was then normalized using min–max scaling. Subsequently, the two feature sets were fused by directly concatenating the two feature vectors, resulting in a 1051-dimensional feature vector that was used as the input to the classifier.

In the design of the classifier, we introduced an attention mechanism in the hidden layer to enhance the model’s predictive performance. Specifically, for the input features $\mathrm{X}=\left[{x}_1,{x}_2,\dots, {x}_{\mathrm{n}}\right]$, we assigned a weight $\mathrm{W}=\left[{w}_1,{w}_2,\dots, {w}_{\mathrm{n}}\right]$to each feature and calculated the weighted sum $\mathrm{Z}$:


(10) \begin{equation*} \boldsymbol{Z}=\boldsymbol{sigmoid}\left(\sum_{\boldsymbol{i}=\mathbf{1}}^{\boldsymbol{n}}\kern0.1em {\boldsymbol{w}}_{\boldsymbol{i}}\cdotp{\boldsymbol{x}}_{\boldsymbol{i}}\right) \end{equation*}

Each feature was then multiplied by the weighted sum, resulting in ${\mathrm{H}}_i$:


(11) \begin{equation*} {\boldsymbol{H}}_{\boldsymbol{i}}={\boldsymbol{x}}_{\boldsymbol{i}}\cdotp \boldsymbol{Z} \end{equation*}

These weighted features were passed to the subsequent fully connected layers. In the hidden layer, we applied the ReLU activation function $f\left({\mathrm{H}}_i\right)$ to introduce nonlinearity:


(12) \begin{equation*} \boldsymbol{f}\left({\boldsymbol{H}}_{\boldsymbol{i}}\right)=\boldsymbol{\max}\left(\mathbf{0},{\boldsymbol{H}}_{\boldsymbol{i}}\right) \end{equation*}

At the output layer, the Sigmoid activation function $\mathrm{\sigma} \left(\mathrm{y}\right)$ was used to obtain the probability of Kla:


(13) \begin{equation*} \boldsymbol{\sigma} \left(\boldsymbol{y}\right)=\frac{\mathbf{1}}{\mathbf{1}+{\boldsymbol{e}}^{-\boldsymbol{y}}} \end{equation*}

Additionally, we incorporated the dropout technique in the model. Dropout works by randomly setting a portion of neuron outputs to zero during the training process, which helps prevent overfitting and improves the generalization ability of the model.

### Feature importance ranking

In this study, feature importance ranking was performed through the following steps: First, the trained PCBert-Kla model was used to predict on an independent external validation set, and the baseline area under the curve (AUC) value was calculated. Then, for each physicochemical feature, we randomly shuffled the values of that feature column in the validation set while keeping the other features unchanged, and re-entered the model to compute the AUC value after shuffling. The importance of each feature was measured by the absolute difference between the baseline AUC and the shuffled AUC. We calculated the AUC change for each fold and summed the AUC changes across all five folds. Finally, based on the total sum of the absolute AUC changes for each feature, we ranked all the physicochemical features.

### Parameter setting

Our hyperparameters were set as follows: 30 training epochs, a batch size of 4, a learning rate of 0.003, the optimizer used was stochastic gradient descent, and the loss function was binary cross-entropy loss. The fully connected layer includes two hidden layers, with 32 neurons in the first layer and eight neurons in the second layer. The attention mechanism is implemented in the first hidden layer. There are two dropout parameters set to 0.1 and 0.3. The first dropout is applied before the first hidden layer, and the second one is applied after the attention mechanism and after the second hidden layer. Additionally, five-fold cross-validation was used, and early stopping was applied based on the highest validation accuracy.

### Evaluation metrics

In this study, we employed a series of commonly used machine learning evaluation methods to comprehensively assess model performance. These methods include ACC, precision (Pre), recall (Rec), F1-score (F1), Matthews correlation coefficient (MCC), and specificity (SP) [44–46]. Additionally, we evaluated the classification capability of the model by plotting receiver operating characteristic (ROC) curves and precision-recall (PR) curves, and calculated the AUC and area under the PR curve (AUPRC), respectively [5, 47–49]. The specific formulas for these evaluation metrics are as follows:


(14) \begin{equation*} \boldsymbol{Pre}=\frac{\boldsymbol{TP}}{\boldsymbol{TP}+\boldsymbol{FP}} \end{equation*}

(15) \begin{equation*} \boldsymbol{Rec}=\frac{\boldsymbol{TP}}{\boldsymbol{TP}+\boldsymbol{FN}} \end{equation*}

(16) \begin{equation*} \boldsymbol{ACC}=\frac{\boldsymbol{TP}+\boldsymbol{TN}}{\boldsymbol{TP}+\boldsymbol{FP}+\boldsymbol{TN}+\boldsymbol{FN}} \end{equation*}

(17) \begin{equation*} \boldsymbol{F}\mathbf{1}=\frac{\mathbf{2}\boldsymbol{PreRec}}{\boldsymbol{Pre}+\boldsymbol{Rec}} \end{equation*}

(18) \begin{equation*} \boldsymbol{MCC}=\frac{\boldsymbol{TP}\times \boldsymbol{TN}-\boldsymbol{FP}\times \boldsymbol{FN}}{\sqrt{\left(\boldsymbol{TP}+\boldsymbol{FP}\right)\left(\boldsymbol{TN}+\boldsymbol{FN}\right)\left(\boldsymbol{TP}+\boldsymbol{FN}\right)\left(\boldsymbol{TN}+\boldsymbol{FP}\right)}} \end{equation*}

(19) \begin{equation*} \boldsymbol{SP}=\frac{\boldsymbol{TN}}{\boldsymbol{TN}+\boldsymbol{FP}} \end{equation*}

where, TP represents the number of correctly identified positive samples, TN denotes the number of correctly classified negative samples, FP refers to the number of falsely predicted positive samples, and FN indicates the number of misclassified negative samples [50–56].

## Results

### Validation of the PCBert-Kla model

**Figure 2:**
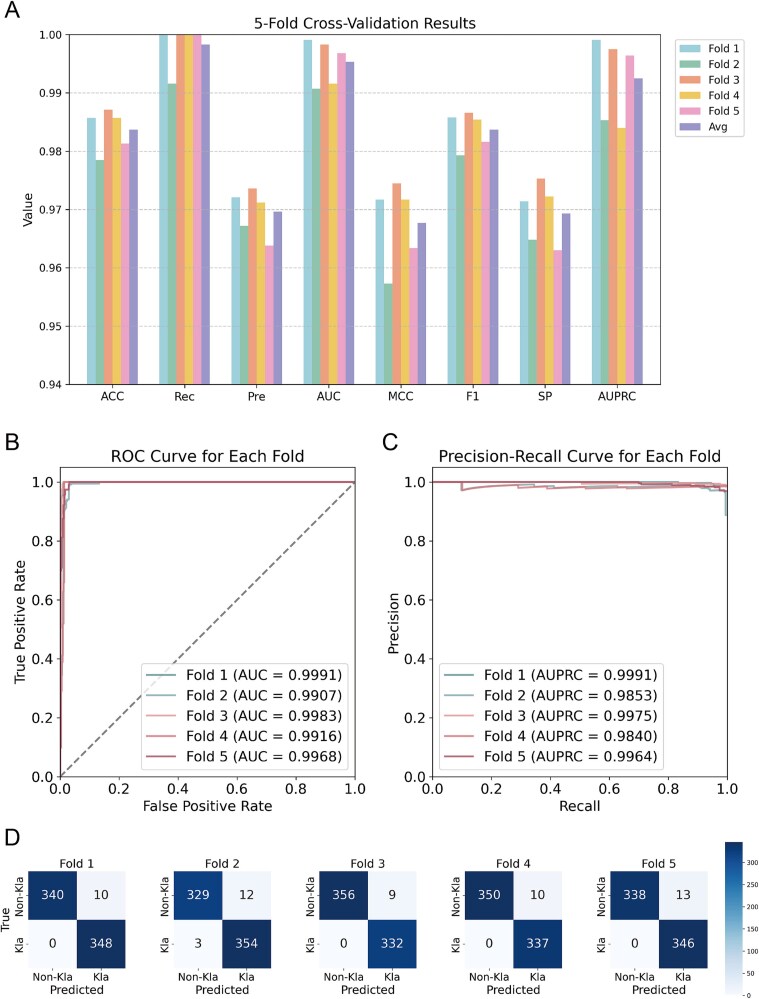
Performance of PCBert-Kla in Kla site prediction. (A) Performance metrics of PCBert-Kla in five-fold cross-validation. (B) ROC curve performance of PCBert-Kla in five-fold cross-validation. (C) PRC curve performance of PCBert-Kla in five-fold cross-validation. (D) The confusion matrix for PCBert-Kla’s five-fold cross-validation.

PCBert-Kla fuses the deep features, which were extracted from ProtBert with various physicochemical properties, creating a rich feature space. Through five-fold cross-validation [57, 58], PCBert-Kla demonstrated robust and superior performance across multiple metrics. It achieved an average ACC of 0.9837, Rec of 0.9983, Pre of 0.9696, AUC of 0.9953, MCC of 0.9677, F1 of 0.9837, SP of 0.9693, and AUPRC of 0.9925 (Fig. 2A, Table 1). These results confirmed PCBert-Kla’s capability to effectively identify Kla modification sites by leveraging comprehensive features. Additionally, its performance in five-fold cross-validation consistently validated its reliability and robustness (Fig. 2B–D, Table 1).

**Table 1:** Performance metrics of PCBert-Kla on the training dataset with five-fold cross-validation.

Fold	ACC	Rec	Pre	AUC	MCC	F1	SP	AUPRC
Fold 1	0.9857	1.0000	0.9721	0.9991	0.9717	0.9858	0.9714	0.9991
Fold 2	0.9785	0.9916	0.9672	0.9907	0.9573	0.9793	0.9648	0.9853
Fold 3	0.9871	1.0000	0.9736	0.9983	0.9745	0.9866	0.9753	0.9975
Fold 4	0.9857	1.0000	0.9712	0.9916	0.9717	0.9854	0.9722	0.9840
Fold 5	0.9813	1.0000	0.9638	0.9968	0.9634	0.9816	0.9630	0.9964
Avg	0.9837	0.9983	0.9696	0.9953	0.9677	0.9837	0.9693	0.9925

### Ablation experiment validation

**Figure 3:**
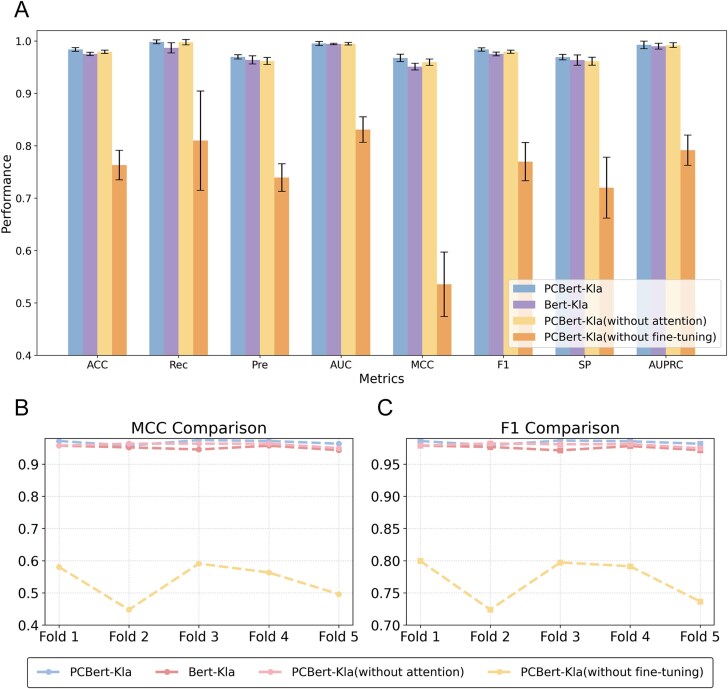
Performance comparison of PCBert-Kla, Bert-Kla, PCBert-Kla (without attention), and PCBert-Kla (without fine-tuning) in Kla site prediction. (A) Comparison of key classification metrics for PCBert-Kla, Bert-Kla, PCBert-Kla (without attention), and PCBert-Kla (without fine-tuning). (B) Comparison of the MCC of the four models. (C) Comparison of the F1 of the four models.

To evaluate the contribution of physicochemical properties, we compared PCBert-Kla with Bert-Kla, which relies solely on deep features extracted from ProtBert. The results showed that PCBert-Kla outperformed Bert-Kla across all metrics (Table S2). Specifically, compared to Bert-Kla, PCBert-Kla showed an improvement of 0.86% in ACC, 1.14% in Rec, 0.59% in Pre, 0.10% in AUC, 1.76% in MCC, 0.86% in F1, 0.59% in SP, and 0.24% in AUPRC. This improvement demonstrates that incorporating physicochemical properties enhances the prediction of the model (Fig. 3A, Table S3). PCBert-Kla outperforms Bert-Kla in MCC, F1, and each fold of the cross-validation, which consistently outperforms Bert-Kla (Fig. 3B and C), further demonstrating the superior robustness of PCBert-Kla.

To further evaluate the effectiveness of the attention mechanism improvement in the fully connected layer and the fine-tuning strategy, we compared PCBert-Kla with two variants while keeping the training strategy and other architectures unchanged: one with the attention mechanism removed from the fully connected layer [referred to as PCBert-Kla (without attention)] and another without fine-tuning ProtBert [referred to as PCBert-Kla (without fine-tuning)]. The results show that PCBert-Kla slightly outperforms PCBert-Kla (without attention) on all metrics and outperforms PCBert-Kla (without fine-tuning) (Table S2). Specifically, compared to PCBert-Kla (without attention) ACC improved by 0.45%, Rec by 0.06%, Pre by 0.80%, AUC by 0.06%, MCC by 0.87%, F1 by 0.43%, SP by 0.81%, and AUPRC by 0.04%. Compared to PCBert-Kla (without fine-tuning), ACC increased by 28.91%, Rec by 23.28%, Pre by 31.17%, AUC by 19.80%, MCC by 80.68%, F1 by 27.80%, SP by 34.64%, and AUPRC by 25.39% (Fig. 3A, Table S3). These results clearly demonstrate that the attention mechanism enhancement in the fully connected layer effectively boosts model performance, and fine-tuning ProtBert improves the model’s adaptability to the task, leading to a substantial increase in predictive accuracy.

**Figure 4:**
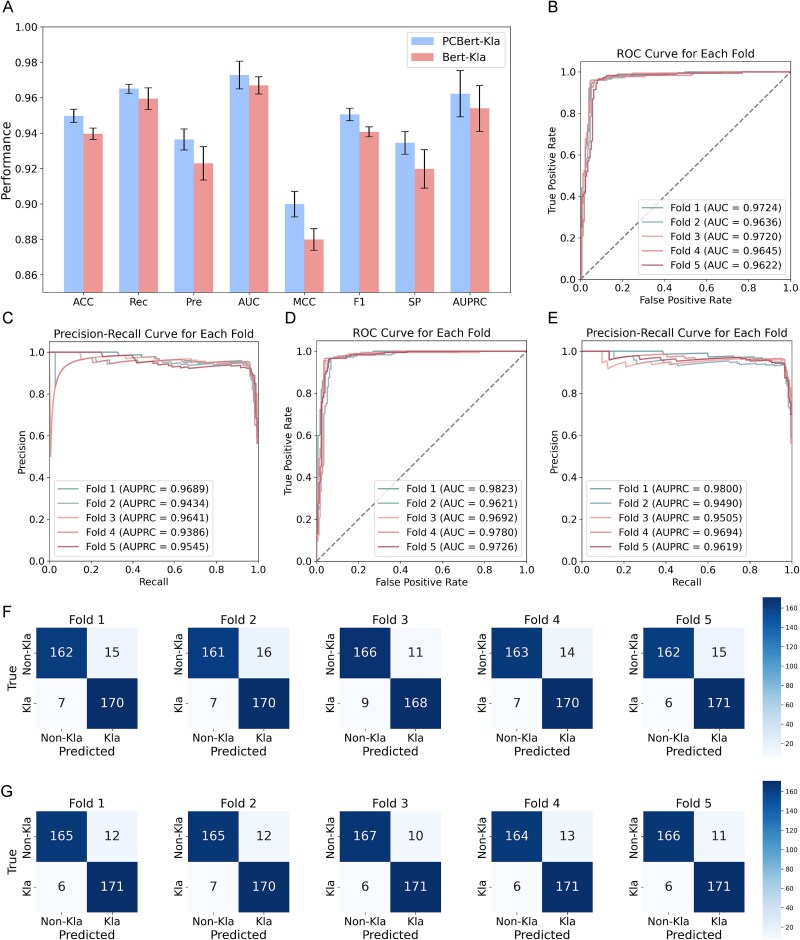
Performance evaluation of Bert-Kla and PCBert-Kla on an independent external dataset. (A) Comparison of key classification metrics for Bert-Kla and PCBert-Kla on the independent dataset. (B) Performance of Bert-Kla’s ROC curve in five-fold cross-validation. (C) Performance of Bert-Kla’s PRC curve in five-fold cross-validation. (D) Performance of PCBert-Kla’s ROC curve in five-fold cross-validation. (E) Performance of PCBert-Kla’s PRC curve in five-fold cross-validation. (F) The confusion matrix for Bert-Kla's five-fold cross-validation. (G) The confusion matrix for PCBert-Kla’s five-fold cross-validation.

### Validation of ProtBert’s applicability in Kla prediction

To further validate the effectiveness of the ProtBert model, we replaced its core pretrained model ProtBert with ESM2 and conducted experiments under the same conditions. The results demonstrated that PCBert-Kla outperformed the ESM2-based variant across multiple performance metrics. Specifically, compared to the model integrating ESM2 with physicochemical features, PCBert-Kla achieved improvements of 0.71% in ACC, 1.51% in Pre, 1.37% in MCC, 0.68% in F1, 1.60% in SP, and 0.05% in AUPRC (Fig. S2, Table S4). These findings suggest that ProtBert is more suitable than ESM2 for the Kla site prediction task in this study.

**Figure 5:**
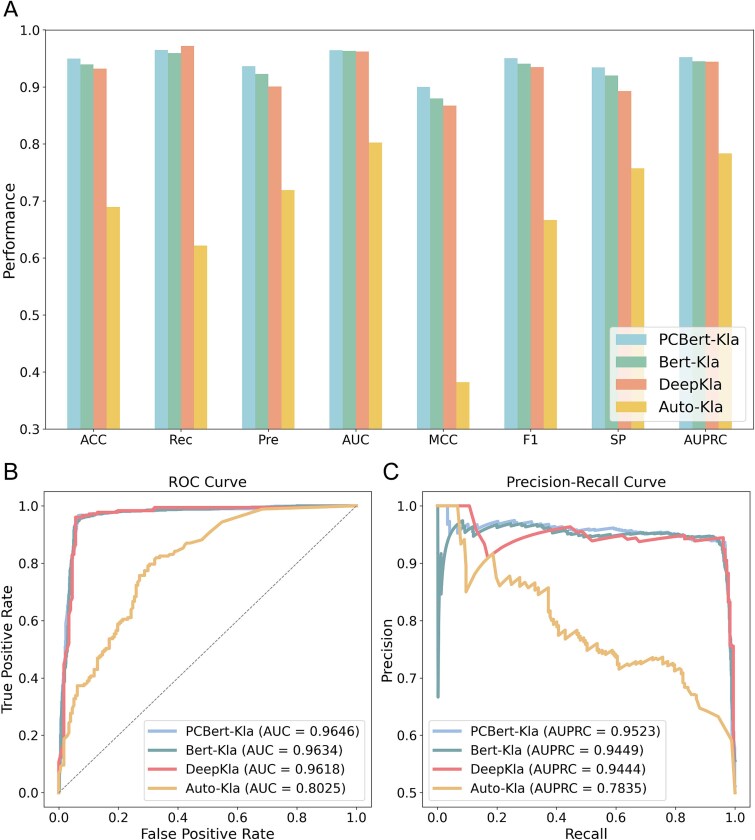
Performance comparison of PCBert-Kla, Bert-Kla, DeepKla, and auto-Kla on the independent test set. (A) Performance comparison of the four models. (B) Comparison of the ROC curves of the four models. (C) Comparison of the PRC curves of the four models.

### Physicochemical feature importance ranking

Based on the feature importance ranking, we found significant differences in the impact of various physicochemical features on the prediction of Kla sites. Net charge was ranked as the most important feature, indicating that the charge properties of proteins play a crucial role in identifying Kla sites. Other important features include E, A, and V, with the proportions of these amino acids contributing significantly to the prediction of Kla sites. Additionally, the helix structure and isoelectric point also showed high importance. In contrast, molecular weight and hydrophobicity contributed less to the model (Table S5).

### Independent external evaluation

To assess the model generalization capabilities in real-world applications, we evaluated them using an independent external dataset. Independent datasets are essential for determining the robustness and generalizability of classifiers. The experimental results demonstrated that PCBert-Kla and Bert-Kla achieved excellent performance across all metrics, with PCBert-Kla outperforming Bert-Kla in every measure (Table S6). Specifically, PCBert-Kla attained ACC of 0.9497, Rec of 0.9650, Pre of 0.9364, AUC of 0.9728, MCC of 0.8999, F1 of 0.9505, SP of 0.9345, and AUPRC of 0.9622 (Fig. 4A, Table S7). Incorporating physicochemical properties enhanced the model’s predictive power and generalization ability. Additionally, PCBert-Kla and Bert-Kla performed exceptionally well in five-fold cross-validation, with PCBert-Kla outperforming Bert-Kla in every fold (Fig. 4B–G, Table S7). This further validates PCBert-Kla’s robustness in predicting Kla sites on new data.

### Comparison with the latest methods

In this experiment, we compared the performance of our models with the advanced algorithmic models DeepKla and Auto-Kla using data from an independent test set. The results were obtained through their publicly available web services. Both PCBert-Kla and Bert-Kla outperformed the existing models across multiple performance metrics. Specifically, PCBert-Kla achieved the highest ACC of 0.9497, followed by Bert-Kla (0.9396), DeepKla (0.9322), and Auto-Kla (0.6893). Additionally, compared to DeepKla, PCBert-Kla showed improvements in Pre by 3.99%, AUC by 0.29%, MCC by 3.78%, F1 by 1.68%, SP by 4.68%, and AUPRC by 0.84% compared to DeepKla (Fig. 5A, Table 2). Meanwhile, our evaluation shows that PCBert-Kla maintains high inference speed even under high-performance conditions (Table S8). These results demonstrate that the PCBert-Kla model outperforms existing methods in prediction performance, highlighting its advantages and potential for practical applications (Fig. 5B and C, Table 2).

**Table 2:** Performance metrics of the four models.

Model	ACC	Rec	Pre	AUC	MCC	F1	SP	AUPRC
Auto-Kla	0.6893	0.6215	0.7190	0.8025	0.3821	0.6667	0.7571	0.7835
DeepKla	0.9322	**0.9718**	0.9005	0.9618	0.8671	0.9348	0.8927	0.9444
Bert-Kla	0.9396	0.9594	0.9229	0.9634	0.8799	0.9407	0.9198	0.9449
PCBert-Kla	**0.9497**	0.9650	**0.9364**	**0.9646**	**0.8999**	**0.9505**	**0.9345**	**0.9523**

**Note:** Bold text indicates the best-performing classifier. The AUC and AUPRC values for Bert-Kla and PCBert-Kla were obtained by aggregating the results from five-fold cross-validation, plotting the combined curves, and calculating the AUCs.

### Model interpretation

**Figure 6:**
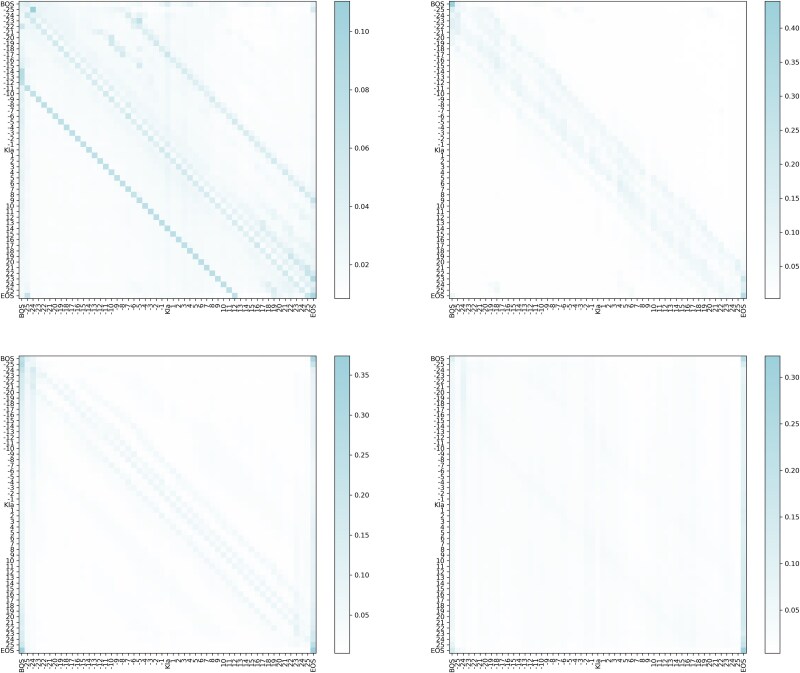
Model interpretation heatmap.

To gain a deeper understanding of how the PCBert-Kla model works, we computed four average attention maps based on all positive samples from the training set using the ProtBert module. Analyzing these maps, we found that, in addition to the beginning of sequence and end of sequence tokens, which exhibited higher attention weights, the model also highlighted significant correlations between each amino acid and its neighboring amino acids. Specifically, each amino acid showed a strong relationship with the adjacent amino acids on either side, indicating that the local sequence context plays a crucial role in predicting Kla sites. Furthermore, we observed that amino acid X has a particularly strong correlation with the amino acid at position X-14, suggesting that structural features might be critical for identifying Kla modification sites (Fig. 6).

### Web server

To facilitate the broader application of PCBert-Kla, we have developed a free online platform (http://pcbert-kla.lin-group.cn/). This platform provides convenient prediction services, supporting two modes of data submission: manual input of amino acid sequences or direct upload of FASTA files. In addition, the platform offers functionalities for exporting prediction results and downloading the datasets associated with this study, thereby enabling users to perform further analysis and research.

## Conclusion

PCBert-Kla model was constructed on the pretrained protein language model ProtBert to improve the accuracy and efficiency of Kla site prediction in proteins. It fully utilizes its powerful feature extraction capabilities, achieving outstanding results across multiple performance metrics. This result highlighted the significant potential of protein large language models in capturing complex PTM patterns from protein sequences. We integrated various physicochemical properties, including molecular weight, isoelectric point, amino acid composition, secondary structure content, hydrophobicity, and net charge. The feature fusion resulted in significant improvements across all performance metrics. Compared to existing models like DeepKla and Auto-Kla, PCBert-Kla outperforms them on multiple evaluation metrics, demonstrating its strong discriminative power and robustness in distinguishing Kla from non-Kla sites. The excellent performance of PCBert-Kla on independent external datasets further validates its generalization capability and practical applicability in real-world scenarios. Additionally, the model analysis shows that local sequence context plays a crucial role in predicting Kla sites. These results highlighted the significant potential of protein large language models in capturing complex PTM patterns from protein sequences.

PCBert-Kla effectively streamlines the process of Kla site identification, offering a new perspective for understanding the regulatory mechanisms of PTMs, and holds promise for facilitating the development of targeted therapeutic strategies. Nonetheless, this study has certain limitations. While PCBert-Kla integrates multiple physicochemical features and demonstrates strong performance across various evaluation metrics, it essentially functions as a “black-box” model. The lack of transparency in its internal decision-making process limits its interpretability. This limitation constrains the model’s utility in uncovering the biological mechanisms underlying Kla modifications and poses challenges for the biological interpretation of its predictions. Future research should focus on enhancing model interpretability to further improve its practical value in life science research.

**Key Points**

- A novel deep learning model, PCBert-Kla, based on ProtBert, is proposed for identifying lysine lactylation (Kla) sites in proteins by extracting deep features from protein sequences.
- The model integrates a variety of physicochemical properties of proteins, including molecular weight, isoelectric point, amino acid composition, secondary structure content, hydrophobicity, and net charge, to enhance the prediction capability.
- An attention mechanism in the fully connected layers allows the model to automatically select the most relevant features for Kla site prediction, improving accuracy.
- PCBert-Kla demonstrates exceptional accuracy and generalization capability in identifying Kla sites, outperforming existing methods in terms of predictive performance.

## Supplementary Material

bbaf615

## Data Availability

The source code has been uploaded to GitHub and can be accessed at: https://github.com/ZhangHongqi215/PCBert-Kla. The datasets have been uploaded to GitHub and can be accessed at: https://github.com/ZhangHongqi215/PCBert-Kla/tree/main/data.
